# Cephalopod RNA recoding: linking protein plasticity to thermal adaptation and catalyzing beyond-species protein characterization

**DOI:** 10.1038/s41392-023-01632-z

**Published:** 2023-10-16

**Authors:** Junyi Wang, Guoping Li, Min Wu

**Affiliations:** 1 Laboratory of Allergy and Precision Medicine, Department of Pulmonary and Critical Care Medicine, Chengdu Institute of Respiratory Health, The Third People’s Hospital of Chengdu, Affiliated Hospital of Southwest Jiaotong University, Chengdu, China; 2 State Key Laboratory of Quality Research in Chinese Medicine, Macau University of Science and Technology, Taipa, Macau SAR, China; 3 Wenzhou Institute, University of Chinese Academy of Sciences, 325000 Wenzhou, Zhejiang China

**Keywords:** Epigenetics, Epigenetics

**Fig. 1:**
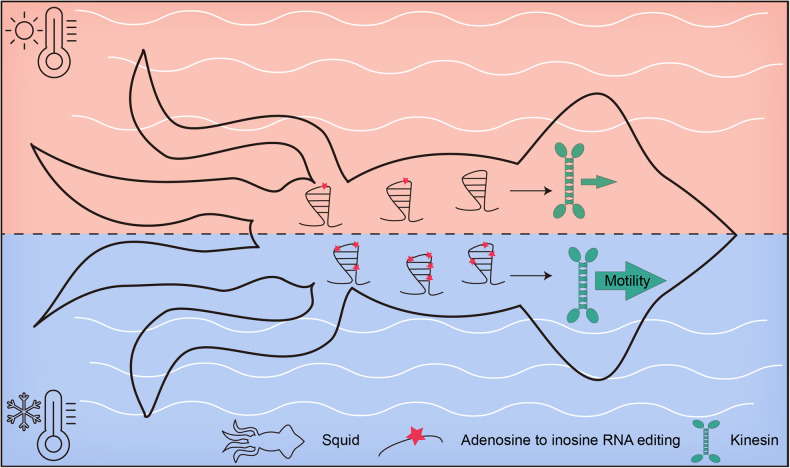
Diagram showing that squids regulate the function of microtubule motor protein kinesin through RNA recoding in response to ocean temperature fluctuations

A recent publication in *Cell* by Rangan et al. highlights the remarkable ability of squids to dynamically regulate the function of microtubule motor proteins through RNA recoding in response to ocean temperature fluctuations (Fig. 1). This study not only provides valuable insights into the crucial role of RNA recoding in generating protein plasticity for adapting to ever-changing environmental conditions but also presents an unprecedented methodology facilitating characterization of conserved non-cephalopod proteins, particularly in the realm of human health and disease.^1^

Adenosine to inosine RNA editing introduces non-synonymous codon changes in coding regions, which are referred to as “recoding,” thereby leading to the production of protein variants with altered amino acid compositions.^2^ Nevertheless, the functional roles of recoding remain largely unexplored. Cephalopods, including squid, octopus, and cuttlefish, exhibit an astonishing prevalence of RNA recoding, rendering them an optimal model system to investigate this intricate process and underlying mechanisms.^3^

Through a GO analysis of edited and unedited proteins derived from *Doryteuthis pealeii*, Rangan et al. unveiled a notable enrichment of edited proteins associated with transport and localization processes.^1^ Within these processes, microtubule motor proteins, such as kinesin and dynein, which are highly conserved and well-characterized, were found to undergo extensive recoding, presenting an exemplification to study the impact of recoding on protein function.^4^ Further, the researchers conducted RNA-seq on the stellate ganglion and optic lobe of *Doryteuthis pealeii*, subsequently revealing the existence of tissue-specific combinations of recoding sites, thereby giving rise to kinesin variants tailored for specific tissues.^1^ Employing single-molecule assays (SMA), they observed significant variations in the motility properties of these variants across different tissues. Notably, a unique variant localized in the optic lobe exhibited reduced velocity, whereas two variants exclusive to the stellate ganglion showed enhanced velocity.^1^ These observations demonstrate the capacity of RNA recoding in distinct tissues to produce kinesin variants with distinct motility characteristics, which aligns with the concept that RNA editing exhibits tissue-specific patterns.^5^

Marine organisms often face substantial temperature variations, influenced by factors including tides, thermoclines, and seasons. These fluctuations impose physiological challenges upon poikilotherms, particularly within their nervous systems.^3^ Could RNA recoding serve as a mechanism through which squid modulates kinesin motility in varying ocean temperatures? Rangan and colleagues conducted an experiment involving *Doryteuthis opalescens*, exposing their hatchlings to a temperature range of 6 °C to 20 °C, which encompasses the extremes experienced by these creatures in their natural habitat.^1^ The study revealed a significant increase in the degree of kinesin RNA recoding as the water temperature decreased. Through individual kinesin transcript sequencing, the researchers further discovered a higher abundance of recoded kinesin variants in colder seawater, while warmer seawater favored unedited kinesin forms. SMA showed that kinesin variants specific for animals exposed to cold seawater exhibited longer run distances and increased microtubule landing rates at 8 °C, surpassing the performance of unedited kinesin, albeit with a slight reduction in velocity.^1^ Yet, a question that arises is the extent to which these findings are applicable in natural environments, or barely in laboratory settings, which necessitates further investigation.

Interestingly, an article by Birk et al. published alongside Rangan’s work in the same issue of *Cell* also revealed that temperature fluctuations in seawater trigger extensive reconfigurations of the neural proteome in *Octopus bimaculoides* through RNA editing, affecting over 13,000 codons and modifying crucial proteins involved in neural processes.^3^ The cold-induced recoding of Octopus kinesin resulted in reduced velocity, shorter run lengths, and an increased propensity for remaining stationary compared to the wild-type version, regardless of whether the temperatures are warm or cold.^3^ Overall, these findings suggest that the recoding of cephalopod kinesin plays a regulatory role in modulating motility characteristics in response to temperature fluctuations.

An intriguing inquiry arises concerning the utilization of the cephalopod editome for interrogating the function of homologs. Taking kinesin as a case in point, Rangan et al. utilized editome data and showed that kinesin exhibited 65% amino acid sequence similarity between cephalopods and humans.^1^ It is worth noting that the majority of recoding sites occur in amino acids that have not been previously demonstrated with functional importance, indicating that cephalopod recoding sites may shed light on residues and substitutions that cannot be readily anticipated using other methodologies. To explore this further, they concentrated on conserved sites of K560 human kinesin motor domains, which show recoding in a minimum of 2% of RNAs across any cephalopod species. This analysis pinpointed a set of 10 recoding site substitutions, impacting 6 amino acids within human kinesin. Subsequent SMA confirmed that the majority of these substitutions had a significant impact on the motility of human kinesin. Importantly, unlike mutations associated with human diseases, the substitutions guided by cephalopods did not eliminate processivity but rather enhanced run distance or velocity.^1^ Further investigations focused on five amino acids that are recoded in cephalopods and their influence on the functionality of human kinesin. The substitution Tyr77Cys resulted in increased run distance without affecting velocity, corresponding with a role in stabilizing neck linker docking. Asn117Asp mutation increased run distance but decreased velocity, and Asn117Gly attenuated both parameters, while Asn117Ser had minimal effects. Kys67Glu slightly reduced run distance, whereas Kys67Arg led to a decrease in velocity and extended run distance. Moreover, Lys281Arg slightly augmented run distance, and Lys166Glu diminished it, consistent with their presumptive roles in stabilizing microtubule interactions.^1^ These studies show the diverse functionality and complexity of recoding sites. Additionally, Rangan et al. assessed the influence of recoding site substitutions on yeast dynein motility, which represents another compelling example of the utility of cephalopod recoding sites in guiding the characterization of conserved homologs.^1^

Altogether, this exciting discovery by Rangan et al. exemplifies the capacity of cephalopod editome to elucidate the functionality of proteins beyond the cephalopod species, emphasizing a methodology that enables more efficient identifications of functionally significant amino acid substitutions. The methods and biological indications described in these studies may help explore potential alterations in amino acids that modify protein function in the context of human health and disease, which offers a fresh perspective on therapeutic RNA editing.
